# Applying an Electronic Health Records Data Quality Framework Across Service Sectors: A Case Study of Juvenile Justice System Data

**DOI:** 10.5334/egems.258

**Published:** 2019-07-11

**Authors:** Matthew C. Aalsma, Katherine Schwartz, Konrad A. Haight, G. Roger Jarjoura, Allyson L. Dir

**Affiliations:** 1 Indiana University School of Medicine, US; 2 American Institutes for Research, US

**Keywords:** electronic health records, data quality, juvenile justice system, administrative data, data linking

## Abstract

**Context:**

Integrating electronic health records (EHR) with other sources of administrative data is key to identifying factors affecting the long-term health of traditionally underserved populations, such as individuals involved in the justice system. Linking existing administrative data from multiple sources overcomes many of the limitations of traditional prospective studies of population health, but the linking process assumes high levels of data quality and consistency within administrative data. Studies of EHR, unlike other types of administrative data, have provided guidance to evaluate the utility of big data for population health research.

**Case Description:**

Here, an established EHR data quality framework was applied to identify and describe the potential shortcomings of administrative juvenile justice system data collected by one of four case management systems (CMSs) across 12 counties in a Midwest state. The CMS data were reviewed for logical inconsistencies and compared along the data quality dimensions of plausibility and completeness.

**Major Themes:**

After applying the data quality framework, several patterns of logical inconsistencies within the data were identified. To resolve these inconsistencies, recommendations regarding data entry, review, and extraction are offered.

**Conclusion:**

The recommendations related to achieving quality justice system data can be applied to future efforts to link administrative databases from multiple sources. Increasing trust in administrative data quality related to vulnerable populations ultimately improves knowledge of pressing public health concerns.

## Context

Public health initiatives to understand and improve the wellbeing of vulnerable populations have increasingly relied on administrative data, namely electronic health records (EHR), to overcome barriers to studying health disparities [1,2]. Integrating EHR with other administrative records across service sectors can provide detailed representations of the contextual and longitudinal factors affecting the health of traditionally underserved populations [2]. Data-linking efforts to integrate these records assume a level of data quality and consistency across systems to achieve an accurate picture of population health [3,4]. Yet, there exists little practical guidance for evaluating the usability of administrative records for research purposes from sources other than EHR [2]. We present a case study assessing the quality of administrative data from juvenile justice systems across a Midwestern state. We deliberately applied an EHR data quality framework [4] to review the justice system data and determine its fitness for public health research. Our approach highlights the importance of standard procedures for achieving quality data that facilitate linking administrative records across systems to understand population health.

The benefits of EHR and other sources of administrative data to study population health have been enumerated previously [2,5]. Most notably, incorporating big data – like EHR – into public health research allows for detecting patterns and trajectories of health care utilization and associated outcomes while avoiding the costly nature of conducting prospective longitudinal studies [6,7]. Longitudinal population studies, such as national surveys of health, require coordinated and labor-intensive data collection efforts and sample maintenance. In comparison, administrative data are less vulnerable to study attrition and participant recall bias [5,6], thereby capturing contemporaneous accounts of real-world, health-related events [4]. National health surveys are also liable to neglect sensitive or rare topics without deliberate over-sampling of sub-populations; a recurring difficulty in studying population health has been the recruitment and retention of vulnerable subject populations over time [8]. Individuals involved in the criminal justice system are one such population, and they are largely left out of national health surveys due to their time spent confined in jails or prisons [5,9,10]. Finally, research on justice-involved individuals has only rarely linked administrative data across multiple systems to explore longitudinal health outcomes [11].

Recent population health studies have focused on health disparities (poor health outcomes that disproportionately impact groups along socioeconomic and racial/ethnic minority lines) [12]. Researchers have asserted that exploring the social determinants of health requires considering justice-involved populations [11,13], individuals greatly affected by health disparities [14]. Compared to the general population, justice-involved individuals are at higher risk for health problems, including sexually transmitted diseases and HIV, as well as mental health and substance use disorders [15]. Because many previous studies of individuals in the justice system have been cross-sectional or based on self-report data [14], there have been calls to enrich studies of the health of incarcerated populations over time [13,16]. Incorporating young, adolescent populations into research designs is one way to explore health issues longitudinally among especially vulnerable and understudied groups. Targeting young people as participants makes it possible to record health behaviors as they change over time, and it allows researchers to focus on prediction and prevention. Yet, justice-involved youth have traditionally been excluded from national population health studies, due to their placement in juvenile detention centers, juvenile prisons, or residential treatment facilities [17].

To study – especially on a large scale – the health of vulnerable populations like justice-involved youth, efforts to link EHR with other administrative records across service sectors have been important. Integrating existing databases allows for studies of data that would not be available from any one source [2,6]. For instance, Binswanger and colleagues [18] integrated criminal justice and health databases and determined that drug overdose among prisoners reentering the community yielded the greatest risk of death within the first few weeks of prison release. There have also been recent calls to employ state-collected administrative data to study access to mental health services among vulnerable populations, including those in corrections [19].

Administrative data present their own challenges to conducting quality research. Because administrative data have been collected for internal record-keeping purposes and not for research, they are susceptible to errors and inconsistencies when used for research purposes [9]. This is in addition to natural variation in data collection and data entry practices across settings. Depending on the primary purpose of data, convenience and expediency may trump consistency and reliability across database users [1,20]. For example, databases that employ free-text notes, as opposed to predefined variable fields, may hinder data linking [1]. Mislinked data, in turn, can lead to biased database creation and flawed data interpretation [3]. For example, if individual subjects are represented in multiple databases but their data remain unlinked, individual cases may be interpreted as non-events, rather than as missing data [21]. Therefore, it is imperative to develop data quality standards and data cleaning procedures that can be applied broadly to administrative data of all types and minimize data-linkage errors.

Studies of EHR provide guidance about how to identify and categorize potential pitfalls of administrative data. Kahn and colleagues [4] have suggested a framework that draws on categories of data quality (e.g., data completeness and plausibility) common to other data quality review approaches [22]. While data quality would ideally be assessed against an established gold-standard, the nature of administrative records precludes such comparisons; administrative records are typically kept as the sole documentation of their contents. Kahn and colleagues [4] suggest, in the absence of a gold-standard, judging the fitness of data for data-linking and for research by triangulating from data within a single database – in this case, the juvenile justice system records themselves – to make determinations about data quality.

Kahn and colleagues describe data quality with three primary dimensions: conformance, plausibility, and completeness [4]. All three data quality dimensions identify logical inconsistencies, or data that veer from what is expected or possible. *Data conformance* refers to whether data values follow prescribed field structures or data dictionary definitions. For example, one would expect that a data field for a subject’s zip code would always contain a numeric, rather than text, value. Similarly, in EHR, a single patient should be assigned a single medical record number (MRN) fitting a pre-defined format. Within this context, an unexpected value or value format discovered during a review of data quality would constitute a violation of data conformance [4]. *Data plausibility* refers to whether individual data values are believable, given their relationship to other variables for the same subject. Violations of data plausibility can be temporal or atemporal in nature, but overall reference misalignment of values. For example, a subject must show a record of facility intake before discharge (meeting temporal plausibility), and a subject with a record of admission to a women’s treatment facility would likely display “female” in a field recording subject gender (demonstrating atemporal plausibility). The last data quality domain, *data completeness*, refers to the level or rate of missingness found within a database, without attention to value format or plausibility. Note that completeness only refers to instances when the data point in question is applicable to the subject [4].

## Case Description

For the present study, we applied the framework outlined by Kahn and colleagues [4] to assess the quality of administrative juvenile justice system data extracted from four unique data management systems across 12 counties in a Midwestern state. We focused on data recorded at primary transition points within the justice system; transition points which may have implications for linking these justice system records to EHR and other sources of administrative data. We anticipate that our efforts will inform local juvenile justice system reform initiatives requiring quality data. The case study presented here could also serve as an example of a systematic quality review process for justice data from other systems.

Our data quality review occurred within the context of three statewide juvenile justice system reform efforts: 1) limiting use of detention placements for juvenile offenders through a statewide Juvenile Detention Alternatives Initiative (JDAI) [23], 2) tracking over-involvement of minority (vs. white) youth in the juvenile justice system in response to federal requirements relating to the Disproportionate Minority Contact (DMC) project [24], and 3) initiating mental health screening for all detained youth (MH Project) [25]. Each of these efforts required accurate and reliable justice system data to appropriately identify the scope of the needed reform and to distribute each initiative’s resources accordingly. For example, to achieve JDAI project success, it was imperative to capture a clear picture of juvenile detention rates within each county to know which populations of youth to target for intervention and to track the initiative’s progress over time. The MH Project similarly required an account of how often juveniles were detained to estimate the associated burden on detention center intake staff administering the mental health screens. The DMC project, a federal initiative, required careful reporting of a youth’s status across several specific decision points within the juvenile justice system (e.g., detained, waived to adult courts) [26] to track whether minority youth were disproportionately represented at each stage of the system. In tandem with these justice system reform efforts, our research team helped form the Juvenile Data Evaluation, Quality and Use Improvement Pilot (J-EQUIP) Project to review data quality.

In the studied state, most counties have adopted one of four sophisticated data/case management systems (CMSs) to record administrative processing of youth within the juvenile justice system. Each CMS allows access to real-time data on how youth move through stages of the system, much like how EHR show a patient’s health care process through a series of encounters with health care professionals and settings [27]. For example, EHR would capture how a patient may progress through an emergency department visit to hospital admission, to discharge from inpatient care, and eventually to follow-up outpatient care. Each of the juvenile justice system CMSs would similarly record if a youth was arrested, detained, and then released back to the community. Despite a common purpose, the four justice system CMSs do not share data or a common user interface, further necessitating the quality review process. The J-EQUIP review was thus undertaken with the practical goal of establishing standard operating procedures for data collection and recording to facilitate state and federal reporting requirements.

## Methods

Counties were selected for inclusion in the J-EQUIP project based on geographic variability to achieve a mix of urban, suburban, and rural counties across all four CMSs. For each CMS, three counties were invited to participate in J-EQUIP, resulting in a total of twelve counties. To assess data quality of a defined dataset, we identified seven turning points, or decision points, within the juvenile justice system that should be represented in the administrative data. Each decision point corresponds to a transition or change in a youth’s status within the system. In this way, we could explore a range of potential data quality issues that were directly relevant to local system reform efforts. These decision points also occur across jurisdictions (i.e., counties), making basic data cleaning principles transferable.

*Seven decision points within the juvenile justice system.* The justice system decision points captured by our quality review included the following: diverted, detained, petitioned, adjudicated delinquent, placed on probation, confined, and waived. These points roughly reflect the order in which a youth would experience stages of the juvenile justice system after formal arrest or school-based referral to the system. (Note that, hereafter, “arrest” indicates arrest or referral.) The range of possible court-imposed sanctions at each decision point typically becomes increasingly punitive as an arrested youth moves through the system. *Diverted* refers to any procedure by which an arrested youth is offered treatment services of some kind and avoids further processing in the justice system. Diverted cases are never adjudicated in front of a juvenile court judge. *Detained* means that the youth has been held in a secure, county-managed short-term (days or weeks on average) holding facility for juveniles. Note that, unlike some other decision points, youth can be detained either before or after other points of system processing. *Petitioned* means that a request for adjudication (i.e., a judge’s decision) has been filed with the juvenile court. *Adjudicated delinquent* indicates that the juvenile court has found sufficient evidence to find allegations against the juvenile to be true. *Placed on probation* indicates that the youth has been ordered to meet certain formal supervision requirements of living in the community in lieu of placement outside of the home. For example, a youth may be subject to unscheduled visits by a probation officer or to completing urine drug screens at random intervals. *Confined* refers to a youth being held in a secure, state-managed long-term (several months on average) prison facility as opposed to a juvenile detention center. Finally, *waived* means that a youth no longer falls under the juvenile court’s jurisdiction; the youth’s alleged offenses are thereafter handled in the adult criminal justice system. All the decision points listed above were to be recorded in each CMS for every individual juvenile as *“yes,” “no,”* or *“missing,”* such that all of these fields should contain a response for each arrest in the system. An individual juvenile may have multiple arrests, and data should be recorded for all decision points for each arrest. We used these seven decision points to assess data quality as follows.

Data were gathered for all months during 2013. Data on all arrests referred to juvenile court for case disposition were extracted from the four CMSs in all 12 participating counties. Because an individual youth may have been arrested multiple times within 2013, each arrest is reflected as a separate “case” in the data. Thus, one individual could contribute multiple cases to the data during the study timeframe. After gathering the arrest data for all juveniles, our research team identified problems with data quality across the four CMSs: logical inconsistencies in the data that violated the data quality dimensions of data conformance, completeness, and plausibility.

## Data Quality Measurement

The dimension of *data conformance* was assessed based on whether recorded responses from the seven decision points per case fell within the limited range of response possibilities: “yes,” “no,” or “missing.”

*Data completeness* was simply reported as the percentage of cases in which “missing” was recorded at each given decision point.

To assess *data plausibility*, our research team reviewed the data reported and looked for common logical inconsistencies based on the seven juvenile justice system decision points described earlier. We identified five of the most common cases of inconsistencies. See Table 1 for a detailed description of each logical inconsistency considered to violate data plausibility.

**Table 1:** Logical inconsistencies violating data plausibility.

Inconsistency	Definition and examples

**Inconsistent petition:** yes to diverted and yes to petitioned	If a case is diverted - meaning that the case was dismissed or resolved through “warn and release,” an informal adjustment (written agreement between the juvenile and the Juvenile Court Probation department), or a referral to a treatment program – there should be no petition filed for formal court processing. If the data extraction suggested that a case had been both diverted and petitioned, often the early decision to divert was overturned by the prosecutor, who then filed a petition. In this scenario, the data would be corrected to reflect “no” for diverted and “yes” for petitioned. Another common scenario resulting in an inconsistent petition occurred if the case was originally diverted through an informal adjustment, but a condition of the agreement was violated by the juvenile. Here, the data would be corrected to reflect “yes” for diverted and “no” for petitioned.
**Implausible case:** no to diverted and no to petitioned	For this plausibility error, a case record was incorrect often because the youth was arrested in one county but held in another county’s detention center. Sometimes the inconsistency meant that a youth was being housed in the local detention center for a case in another county (i.e., a courtesy detention). To correct these data errors, these cases would not be included in the total number of cases within a county, since the court processing occurred in another jurisdiction. In other cases, this type of inconsistency alerted our research team to widespread clerical errors that occurred across the county, which required scrutiny of the entire case chronology.
**Excess information:** yes to diverted and yes to subsequent decision points	For the purpose of recording a diverted case, the diversion should be the last decision point. Thus, when the data extracts for a diverted case included information on subsequent decision points, it was likely that subsequent arrests for the same youth were erroneously linked to the original diversion. In most cases, the excess information was not applicable to the diversion in question, meaning that the excess information would apply to separate arrests. In other cases, the diversion field was incorrectly filled, requiring a simple correction.
**Inconsistent waiver:** yes to waived and yes to subsequent decision points	Similar to diversion cases, if the youth is waived to adult court, there should be no further activity in juvenile court related to the same arrest. The data extractions showed that “waived” was the decision point most likely to be incorrectly noted in the CMSs. Data entry corrections resolved these errors.
**Inconsistent adjudication:** no to adjudicated delinquent and yes to later decisions	An adjudicatory hearing in which the arrest was found “not true” is another way to conclude a juvenile case. There should be no formal probation or confinement in a correctional facility if the youth is not adjudicated delinquent. In most of these inconsistent adjudication cases, the error occurred because the adjudication field was left blank, and subsequent decision points accurately reflected the case outcome.

To record inconsistencies, we only recorded the first inconsistency within an individual youth’s court case chronology. Thus, although a single case may have had more than one inconsistency, only one error per case is reported. For example, if a single arrest was noted to have both an inconsistent petition (yes to diverted and yes to petitioned) and an inconsistent waiver (yes to waived and yes to subsequent decisions), only the inconsistent petition would be recorded as an error given the chronological order of petition prior to waiver. We report the percentage of cases for which each inconsistency was the first recorded error within a case. The reason for presenting our findings in this way is that, in a relational database like the CMSs described here, data fields are logically interdependent. One decision point data field containing an error could affect the meaning of responses to many other data fields within a case. Therefore, once a violation of data plausibility is detected, all other decision points must be reviewed to determine the correct case outcomes.

The data were then presented to CMS system representatives to allow for corrections and cleaning. This included identifying missing data elements and exploring court case files to understand common data inconsistencies and make changes based on paper records and juvenile probationer notes. Representatives from the CMSs were then asked to extract the data a second time. We report comparisons of data completeness and data plausibility before and after data cleaning.

## Findings

Characteristics of each case extracted by CMS are presented in Table 2. More than half of all cases reviewed involved white (55.9 percent – 86.4 percent), male youth (62.6 percent – 69.5 percent) older than age 14 (65.1 percent – 73.6 percent). We also report the percentage of cases by severity of the most serious charge associated with the case. The second most severe felony designation (Felony B), for instance, was charged in 1.7 percent – 2.0 percent of cases by CMS. Within each CMS, cases most commonly involved a serious misdemeanor (Misdemeanor A; 21.2 percent – 27.4 percent) or a status offense (12.8 percent – 28.7 percent) as the most severe charge. As reflected in Table 2, CMS 4 did not provide data regarding youth race or charge severity.

**Table 2:** Case Description by CMS (N = 16,013)*.

	CMS 1		CMS 2		CMS 3		CMS 4	

	n = 1,474		n = 870		n = 12,662		n = 1,007	

	n	(%)	n	(%)	n	(%)	n	(%)

**Youth Gender**								
Male	923	(62.6)	604	(69.5)	8,527	(67.3)	669	(66.4)
Female	551	(37.4)	266	(30.5)	4,135	(32.7)	338	(33.6)
**Youth Race/Ethnicity**								
African American/Black	181	(12.3)	63	(7.2)	3,799	(30.0)		
Asian	<10		<10		71	(0.6)		
Hawaiian/Pacific Islander	<10		<10		<10			
Hispanic/Latino	79	(5.3)	25	(2.9)	624	(4.9)		
Native American or Native Alaskan	<10		<10		27	(0.2)		
White	1,172	(79.5)	752	(86.4)	7,080	(55.9)		
Other	30	(2.1)	<10		1,057	(8.3)		
**Youth Age**								
10	21	(1.4)	<10		97	(0.8)	<10	
11	33	(2.2)	14	(1.6)	231	(1.8)	18	(1.8)
12	90	(6.1)	23	(2.7)	503	(4.0)	38	(3.8)
13	157	(10.6)	73	(8.4)	1,011	(8.0)	97	(9.7)
14	215	(14.6)	116	(13.4)	1,790	(14.1)	146	(14.5)
15	314	(21.3)	156	(17.9)	2,475	(19.5)	201	(19.9)
16	330	(22.4)	225	(25.8)	3,186	(25.2)	254	(25.2)
17	300	(20.4)	254	(29.2)	3,206	(25.3)	233	(23.2)
18	15	(1.0)	<10		163	(1.3)	10	(1.0)
*Mean age (SD)*	*15.0*	*(1.7)*	*15.4*	*(1.5)*	*15.3*	*(1.6)*	*15.2*	*(1.6)*
**Arrest Charge Severity (descending)**								
Felony A	<10		<10		<10			
Felony B	28	(1.9)	15	(1.7)	254	(2.0)		
Felony C	61	(4.1)	26	(3.0)	253	(2.0)		
Felony D	336	(22.8)	214	(24.6)	1,616	(12.8)		
Misdemeanor A	312	(21.2)	238	(27.4)	3,132	(24.7)		
Misdemeanor B	206	(14.0)	83	(9.5)	1,805	(14.3)		
Misdemeanor C	101	(6.8)	180	(20.7)	226	(1.8)		
Status Offense	423	(28.7)	111	(12.8)	2,623	(20.7)		
Violation of Probation	<10		<10		2,751	(21.7)		

* Cell values under 10 individuals are not reported to limit possible identification.

Findings related to data completeness and data plausibility are reported in Tables 3 and 4, respectively. Table 3 shows the percentage of cases with missing data at each of the seven decision points by CMS. Missingness varied widely by CMS. Data extractions revealed that CMSs 2 and 3 had virtually no missing data, while CMSs 1 and 4 had significant amounts of missing data at a few of the decision points. All information from CMS 1 regarding confinement and waiver was missing. Roughly a third of all data was missing for adjudication, probation, and confinement in CMS 4. Post data cleaning efforts, missingness within CMS 1 was reduced to 31.3 percent of confinement data and 18.0 percent of waiver data. No post-cleaning data were available for CMS 4.

**Table 3:** Violations of data completeness: Percentage of cases with missing data at each decision point by CMS, pre- and post-data cleaning.

	CMS 1		CMS 2		CMS 3		CMS 4	

	n = 1,474		n = 870		n = 12,662		n = 1,007	

	Pre-	Post-	Pre-	Post-	Pre-	Post-	Pre-	Post-

Diverted	0.0%	0.0%	0.0%	0.0%	0.6%	0.6%	0.0%	
Detained	0.0%	0.0%	0.0%	0.0%	0.0%	0.0%	0.0%	
Petitioned	0.0%	0.0%	0.0%	0.0%	0.3%	0.6%	0.0%	
Adjudicated delinquent	0.0%	0.0%	0.0%	0.0%	0.4%	0.8%	29.7%	
Placed on probation	2.2%	2.2%	0.0%	0.0%	0.4%	0.8%	29.7%	
Confined	100.0%	31.3%	0.0%	0.0%	0.4%	0.8%	29.7%	
Waived	100.0%	18.0%	0.0%	0.0%	0.6%	0.6%	0.0%	

Table 4 reflects the percentage of cases in each CMS exhibiting a specific logical inconsistency that violated data plausibility, both before and after the data cleaning process. Again, the data extracted varied substantially by CMS. The percentage of cases with at least one inconsistency ranged from 12.4 percent – 95.5 percent across the CMSs. Cases from all four CMSs contained an “implausible case” error and an “inconsistent adjudication error,” while “inconsistent waiver” errors were rare in CMSs 1–3. CMS 2 showed the most reduction in data plausibility violations post-data cleaning, though the data cleaning process was successful for CMS 3 and CMS 1 as well. Again, CMS 4 did not provide post-cleaning data.

**Table 4:** Data plausibility violations: Percentage of cases with logical inconsistencies by CMS, pre- and post-data cleaning.

	CMS 1		CMS 2		CMS 3		CMS 4	

	n = 1,474		n = 870		n = 12,662		n = 1,007	

	Pre-	Post-	Pre-	Post-	Pre-	Post-	Pre-	Post-

Inconsistent petition	0.0%	0.0%	24.9%	0.0%	0.0%	0.0%	0.0%	
Implausible case	3.6%	2.0%	20.1%	0.1%	8.4%	1.3%	48.5%	
Excess information	0.7%	1.3%	0.1%	0.0%	0.0%	0.0%	6.4%	
Inconsistent waiver	0.0%	0.1%	0.0%	0.0%	0.0%	0.0%	18.7%	
Inconsistent adjudication	9.9%	10.7%	26.0%	0.0%	4.0%	4.9%	22.4%	
At least one inconsistency	14.2%	14.1%	71.2%	0.1%	12.4%	6.2%	95.5%	

## Major Themes

The patterns of logical inconsistences within the data led our J-EQUIP team to derive principles to consider when drafting standard operating procedures for both data entry and data review. We learned that a first step to improve data quality is cooperation from both the CMS system representatives as well as CMS end users (i.e., those who enter data and review cases through the system). It was important to seek the buy-in of both system representatives and end users as they independently contribute to data quality. For example, significant amounts of data were missing from CMS 1 and 4 upon initial review, but only for specific decision points. CMS 1 was missing all data on confinement and waiver pre-cleaning, and CMS 4 was missing all data for three decision points. Discussions with representatives from these CMSs revealed that end users had varying awareness of the rules for data entry and may not have understood the importance of following existing data entry rules consistently, likely resulting in empty data fields and other errors. Juvenile probation officers were often the end users of the CMSs, but probation officers are no longer involved in a case when a youth is sent to confinement or waived to adult court. Thus, probation officers had little knowledge of data entry rules regarding confinement and waiver-related data fields and had little incentive to fill out those fields after a case was closed. Representatives in CMS 2 counties, which evidenced the most improvement in data plausibility post-cleaning, quickly developed detailed feedback for those completing data entry, suggesting the importance of ongoing communication with end users. The need for regular, thorough training by CMS system representatives, and participation in training by all CMS end users, was a theme throughout the data quality review process.

A second data review theme was that the structure of data entry fields (i.e., “fixed” fields versus open notes fields) and the data entry protocol should align with both the purpose of data collection and the purpose of data extraction and analysis. The primary purpose of a CMS, according to its end users (usually juvenile probation officers), is case management, meaning that data entries are made according to the needs of a youth’s probation officer. As such, some of the decision points appeared to be missing data because those data points were not directly relevant to the probation officers’ use of the system. As previously stated, when a juvenile is confined in the Department of Corrections or waived to adult criminal courts, probation officers are no longer involved in the youth’s case. Because probation officers had no practical need to resolve related data inconsistencies within the CMS, data utility impeded comprehensiveness and completeness. This is a problem common to administrative data [28]. The practical recommendation related to this problem is to identify mandatory data entry fields for all cases within a CMS, such that a user receives an error message if specific fields are left unfilled. At the time when the J-EQUIP project occurred, the CMSs were not designed to flag missing data or common logical inconsistencies, which was one reason why the data quality review process was valuable. It would be optimal if systems could be redesigned to alert users of the inconsistencies as they are entered. If a youth, for instance, is assigned to formal probation but the system also shows that same youth was not adjudicated delinquent, the problem should trigger a requirement to resolve the inconsistency at the time the youth is recorded as placed on probation. In the absence of such redesigns, the J-EQUIP process is an efficient way to identify the most important issues to resolve in a database.

Another data review theme identified relates to the fact that probation officers, the primary users of CMSs, cannot predict how any individual youth’s case will proceed through decision points over an extended period. The CMSs are relational databases, meaning that data entered into one decision point field is often dependent on previous decision point data entries. A complete picture of case processing, therefore, can only be achieved once the case is closed. Thus, the dynamic nature of case processing impacts determinations of overall data quality. Data extraction for data quality review purposes compounds this problem; when aggregated data are extracted from the CMSs for reporting purposes, decision points are typically reflected in separate tables, and the links between decision points become invisible. In other words, because of the dynamic nature of case processing, data extractions lack meaningful evidence of various case processing decisions for any individual case. Unfortunately, the only short-term recommendation to address this issue is to encourage CMS users to regularly review samples of individual cases to identify common violations of data plausibility based on case chronology.

Beyond data entry and review considerations alone, developing a meaningful data extraction process for these relational databases is imperative. To generate the kinds of data reports that support data-driven decision making, it is important to be able to extract data in ways that facilitate telling a coherent and complete story. Some systems provide flexibility to data users, such that they can record the same information using different approaches. Many users, for instance, may utilize comment fields to record narrative descriptions of case outcomes, rather than complete specific data entry fields. Yet, to facilitate the accurate aggregate reporting of the various decisions, it is important that information can be located in discrete locations and are completed for every case. For the present study, due to the practical requirements of ongoing local system reform efforts, we selected a finite number of data fields to extract. However, data extractions performed to assess data quality can themselves be limited by the ways in which the users are entering information into the database. If the data extraction relies on underutilized data fields, then the data extraction may not provide a meaningful subset of information by which to assess data quality. Thus, the developers of the data systems must balance the flexibility of the data entry options with the data processing needs. Through training, those entering data can be encouraged to enter the data in the targeted fields, but doing so necessitates careful and early construction of a data processing plan.

## Challenges and Study Limitations

This data review process highlighted several barriers to achieving quality juvenile justice system data, many of which mirror problems encountered in data quality improvement efforts in EHR and other administrative data systems. These challenges to obtaining data quality may similarly affect linking justice system data to EHR and, ultimately, may impact the utility of these data in public health initiatives. Thus, each of the following issues should be carefully considered in future efforts to conduct reviews of administrative data quality.

First, despite the support of J-EQUIP in the current project, resources and opportunities to support IT and data entry training in the juvenile justice system are lacking, especially in smaller jurisdictions [29]. Many of our suggestions for improving future data quality of the CMSs are contingent upon available funding and personnel. Fortunately, with increases in initiatives to use administrative data for public health research, there are now free online training courses available that could be implemented in jurisdictions to improve data entry and review [29].

Second, we learned that it can be time consuming and inefficient to clean administrative data for research purposes [9]. Our efforts required a thorough understanding of both how a youth might be processed through the justice system and the corresponding rules for data entry to accurately track a youth’s process. As in reviews of EHR data, in order to understand patterns observed in the data, including patterns of inconsistencies and missingness, it is necessary to first learn how the data were generated [30]. This important step to improving data quality can be a tedious and lengthy process for data reviewers who may be unaware of the origin, purpose, or structure of individual administrative datasets.

Third, data definitions may change over time for practical reasons or new performance standards [9]. For example, changes in policy related to waiving a youth from the juvenile to adult system could influence changes in data definitions in CMSs and, in turn, influence the potential for data inconsistencies. Therefore, data definitions and ensuing data inconsistencies warrant continual monitoring.

Lastly, data missingness was a major issue in the current project. We found one CMS was unable to provide follow-up data due to leadership change within the organization, leaving us unable to comment on data quality improvement. This is also true for EHR, as data missingness is perhaps the greatest challenge to applying data to inform public health initiatives [31].

## Conclusion

The findings reported here emphasize that some of the challenges to achieving administrative data quality will apply to future efforts to link justice system data to EHR. We found that, like in other reviews of administrative data [4,27,32], there was substantial inconsistency and missingness within the data. These errors regarding justice system involvement data are problematic for future public health research, as justice system involvement predicts a host of other health risk factors within vulnerable populations [17,32]. Further, it is vitally important to strive for quality juvenile justice system data because involvement in the system is, by definition, time-limited (i.e., until age 18). Thus, early data errors could have potentially long-lasting consequences to youth and when reporting on youth outcomes.

EHR data quality reviews can be a model for preparing other types of administrative data for linking across systems and conducting comprehensive public health research. The present study offers an example of an application of an EHR data review framework to administrative records from several juvenile justice data systems. Applying the framework by Kahn and colleagues [4] to assess both data completeness and data plausibility identified a range of logical inconsistencies that could be relevant to other justice system records. Each logical inconsistency dictated an appropriate solution, which worked to improve data quality for future reviews. By establishing more accurate and reliable justice system data, the chances of successfully linking these data across other administrative systems are likely improved. Ultimately, by increasing trust in data related to some of the most vulnerable populations, we expand our understanding of some of the most pressing public health concerns.
